# Signatures of AAV-2 immunity are enriched in children with severe acute hepatitis of unknown etiology

**DOI:** 10.1126/scitranslmed.adh9917

**Published:** 2023-07-26

**Authors:** Moriah M. Mitchell, Yumei Leng, Suresh Boppana, William J. Britt, Luz Helena Gutierrez Sanchez, Stephen J. Elledge

**Affiliations:** 1 Division of Genetics, Department of Medicine, Howard Hughes Medical Institute, Brigham and Women’s Hospital, Boston, MA 02115, USA; 2 Department of Genetics, Harvard Medical School, Boston, MA 02115, USA; 3 Program in Systems, Synthetic, and Quantitative Biology, Harvard University, Boston, MA 02115, USA; 4 Division of Pediatric Infectious Diseases, Department of Pediatrics, University of Alabama at Birmingham, Birmingham, Alabama, USA; 5 Division of Gastroenterology, Hepatitis, and Nutrition, Department of Pediatrics, University of Alabama at Birmingham, Birmingham, Alabama, USA

## Abstract

Severe acute hepatitis of unknown etiology in children is under investigation in 35 countries. Although several potential etiologic agents have been investigated, a clear cause for the liver damage observed in these cases remains to be identified. Using VirScan, a high throughput antibody profiling technology, we probed the antibody repertoires of nine cases of severe acute hepatitis of unknown etiology treated at Children’s of Alabama and compared their antibody responses to 38 pediatric and 470 adult controls. We report increased adeno-associated dependoparvovirus A (AAV-A) breadth in cases relative to controls and detailed adeno-associated virus 2 (AAV-2) peptide responses that were conserved in 7 of 9 cases but rarely observed in pediatric and adult control. These findings suggest that AAV-2 is a likely etiologic agent of severe acute hepatitis of unknown etiology.

## One-sentence summary

AAV-2-reactive antibodies and evidence of AAV-2 helper virus infection are associated with pediatric severe acute hepatitis of unknown etiology.

## Introduction

Recent reports of acute hepatitis of unknown etiology (AHUE) in children have sparked concern globally with over 1000 probable cases identified since October 2021 (1). In the United States, 372 children were under CDC investigation for acute hepatitis of unknown cause as of October 2022 (2) with 22 associated liver transplants and 14 deaths reported (3). Although overall incidence of pediatric hepatitis cases in the United States may not have increased over pre-pandemic incidence (4), spatiotemporal clustering of cases in Alabama, Scotland, and the Netherlands has prompted the search for a shared etiologic agent (1, 3, 5). Hypotheses under investigation include environmental or toxin exposure, pathogen exposure, superantigen or autoimmune reactions to persistent or previous severe acute respiratory syndrome coronavirus 2 (SARS-CoV-2 infection), and altered response to first adenoviral exposure as a result of delayed initial exposure due to infection control behaviors and isolation brought on by the coronavirus disease 2019 (COVID-19) pandemic (3).

Human adenoviruses (HAdVs), particularly adenovirus F subtypes 40 and 41, are a leading exposure under investigation as a potential trigger of AHUE (6). In contrast to HAdVs B to E, Adenovirus F is well adapted for gastrointestinal tropism as result of structural differences in the capsid fibers which enable stability at low pH (7) and is a leading cause of gastroenteritis in children (8). Although HAdV infection in general has been implicated in some cases of hepatitis, reports of adenovirus associated hepatitis in immunocompetent patients are scarce (9–11). Of the 9 cases of AHUE identified in the initial Alabama cluster in the United States, 8 (89%) tested positive for human adenovirus infection by whole blood quantitative polymerase chain reaction (qPCR) and all five for which subtyping was possible were found to be Adenovirus 41 (6). Adenovirus infection has been detected less frequently in other case series. In a cluster in Scotland, 5 out of 13 children (38%) tested positive for HAdV by PCR testing of throat swab, blood, or stool (5). Only 45% of all children in the United States and 52% of children in Europe under investigation for AHUE were found to be positive for any HAdV where testing was performed (1, 3).

In order to investigate possible viral etiology of AHUE, we employed VirScan, a phage display immunoprecipitation sequencing (PhIP-seq) technology that detects antibody binding to peptides derived from the proteomes of selected pathogens. We studied the anti-viral antibody repertoires of nine patients with AHUE in comparison to pediatric and adult controls. Here, we present evidence of a conserved signature of adeno-associated dependoparvovirus A (AAV-2) immunity in cases of AHUE that was not observed in pediatric or adult controls. These findings point to AAV-2 as a potential etiologic agent for AHUE development.

## Results

### Sample characteristics

Serum isolated from whole blood of 9 patients with severe acute hepatitis of unknown etiology admitted to Children’s of Alabama between October 1, 2021, and February 28, 2022, were analyzed (Table 1). Serum samples collected from 38 healthy children prior to the COVID-19 pandemic were used as pediatric controls. Serum samples obtained from 470 adults with current or prior SARS-CoV-2 infection were used as additional controls.

### Overall breadth of antiviral antibody responses did not differ between cases and pediatric controls.

To explore the hypothesis that AHUE is triggered by atypical immune response due to delayed initial pathogen exposure caused by pandemic infection control measures in early life, we examined the antibody breadth of cases and pediatric control samples collected prior to the COVID-19 pandemic. If delayed initial pathogen exposure played a role in development of AHUE, one would expect to see reduced breadth of antibody response in cases versus controls; however, no difference in overall antibody breadth, measured as the total number of VirScan peptides targeted in a sample, was observed between cases and pediatric controls (p = 0.78 for Welch’s 1-sided t-test). Of the 115,753 peptides in the VirScan library used, samples targeted a mean of 1352 ± 40 peptides. After correcting for multiple testing, the only significant difference in breadth of antibody response at the family level occurred for *Parvoviridae* (p =1.47 × 10^−3^ for Welch’s 1-sided t-test).

**Fig. 1.**
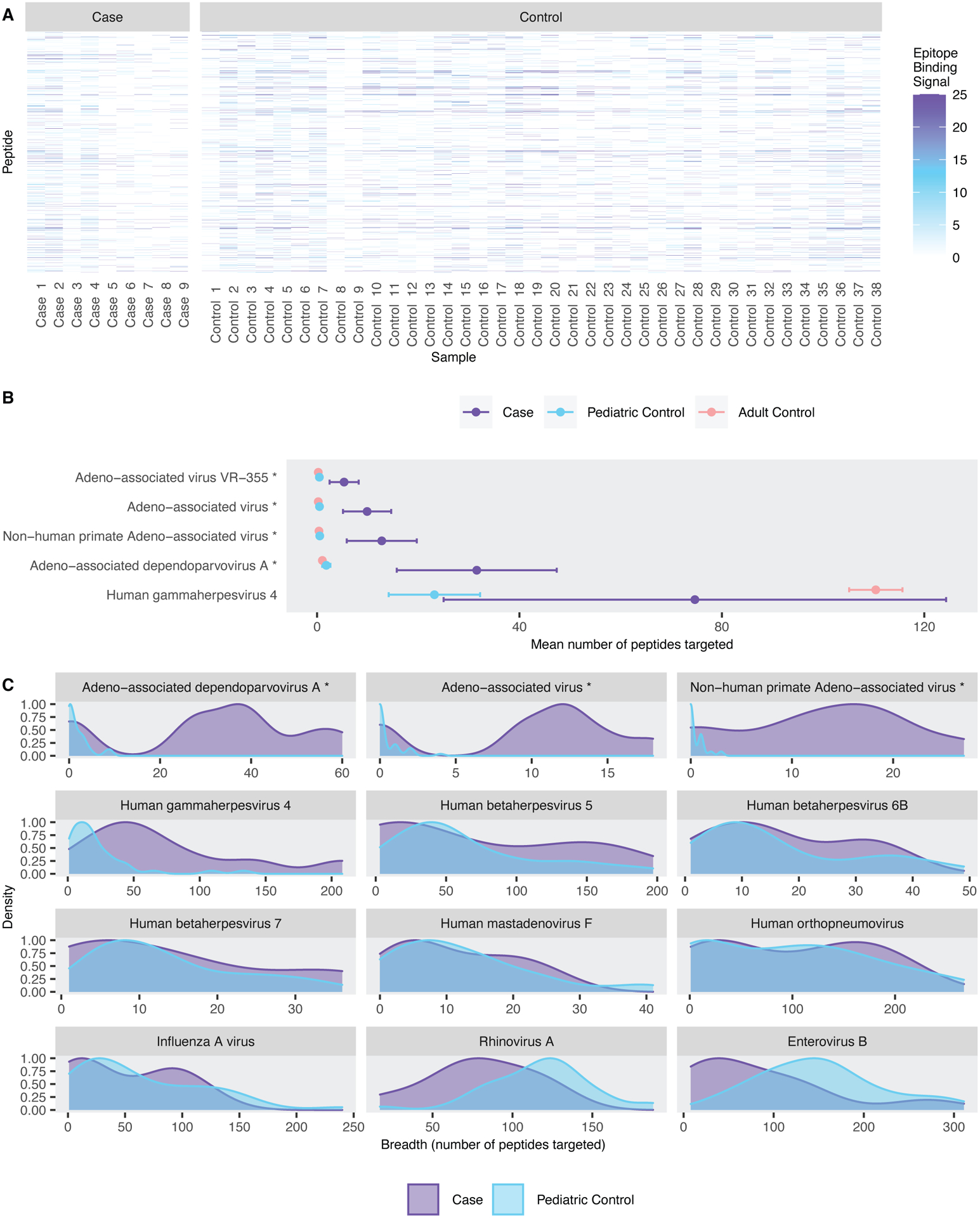
Breadth of AAV-specific antibody responses differed between cases and controls. (**A**) Shown is a heatmap of epitope binding signals (EBSs) for all peptides targeted by cases or pediatric controls (n=18,484). Raw data are available in data files S1 to S2. (**B**) The mean number of species-specific peptides targeted in cases, pediatric controls, and adult controls are shown for pathogens with nominally significant differences (p <0.05) as measured with Welch’s one-sided t-test with 95% confidence interval. Pathogens with statistically significant (FDR=0.05) differences after Benjamini-Hochberg correction are annotated with *. (**C**) Pathogen-specific breadth of antibody response in cases (purple) overlaid on the distribution of breadth in pediatric controls (blue, n=38) is shown for selected common pathogens and AAV’s.

As overall breadth of antibody response was not different between cases and controls, we explored whether differential antibody responses to specific pathogens were observed between cases and controls (Fig. 1A). Although nominally significant differences (Welch’s 1-sided t-test, alpha=0.05) in mean breadth of response between cases and pediatric controls were detected across 5 pathogens (Fig. 1B), only differences in responses to 4 *Parvoviridae* species (Adeno-associated dependoparvovirus A, Adeno-associated virus, Adeno-associated virus VR-355, and non-human primate Adeno-associated virus) were significant after correcting for multiple hypothesis testing [false discovery rate (FDR) = 0.05]. Of 9 cases, 7 appeared to have strong antibody responses to AAVs. Breadth of pathogen-specific response to other common childhood pathogens in cases fell well within the distributions observed in pediatric controls (Fig. 1B and C).

### Epitopes Associated with Severe Acute Hepatitis of Unknown Etiology

**Fig. 2.**
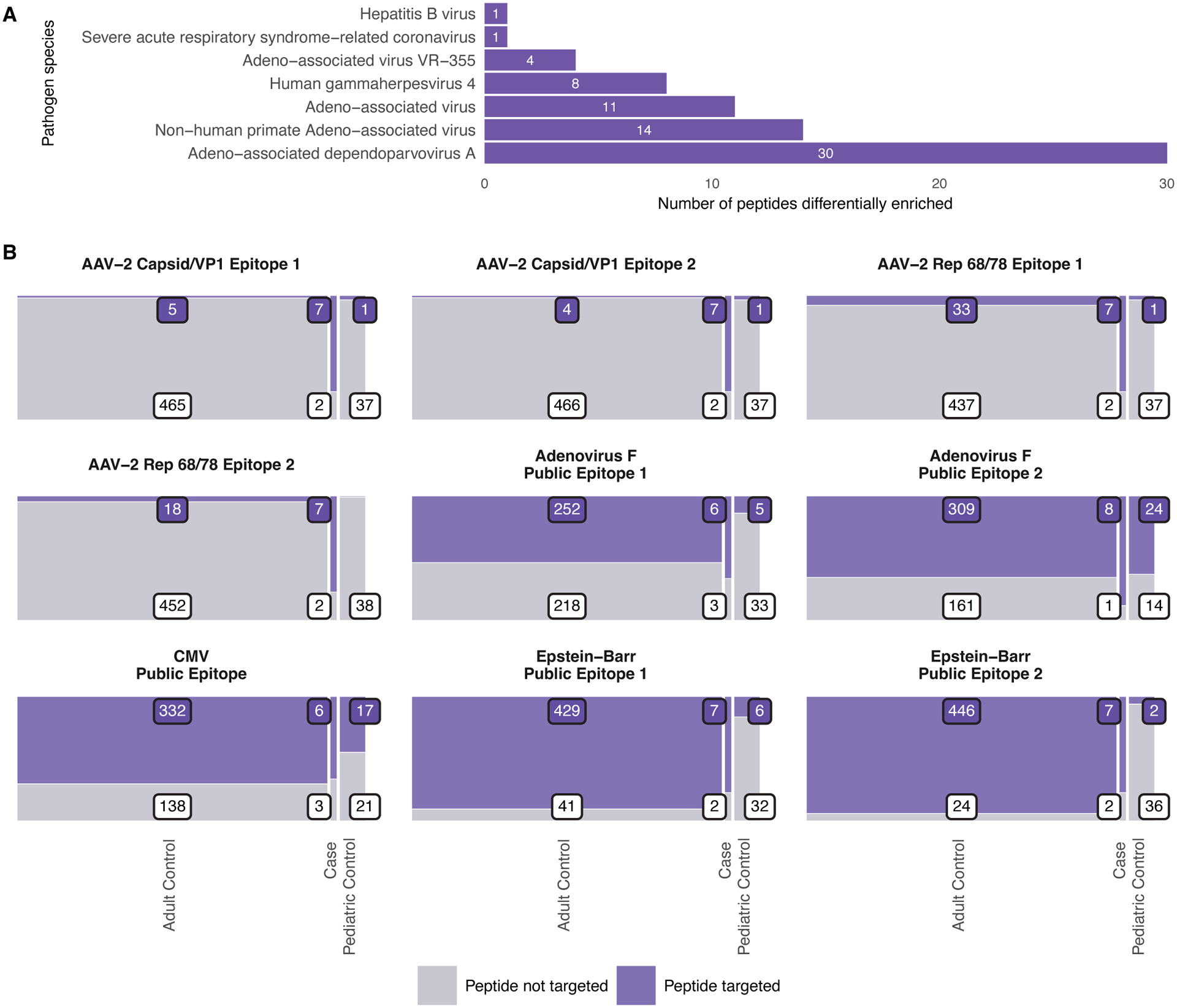
AAV peptides were targeted at increased frequency in cases versus controls. (**A**) Shown is the number of peptides targeted at a significantly higher frequency in cases than pediatric controls (Fisher’s one-way Exact test with Benjamini-Hochberg procedure and FDR of 0.05) by viral species. (**B**) Mosaic plots show reactivity to representative AAV-2 peptides heavily targeted in cases and previously identified herpesvirus, adenovirus, and influenza public epitopes in cases, pediatric controls, and adult controls. The number of individuals that do or do not target each peptide are indicated in boxes on the figure. Peptide-level hit data for all samples and peptides are available in data files S3 to S5.

In order to identify peptides targeted at significantly higher frequency in cases than controls, we used Fisher’s one-way Exact Test and adjusted for multiple tests with the Benjamini-Hochberg Procedure. Sixty-nine peptides from 7 viruses were significantly enriched in cases versus pediatric controls (FDR=0.05) (Fig. 2A). Sixty of these peptides were also significantly (FDR=0.05) enriched in cases versus the adult control cohort in which in which samples were collected at a more similar time and location to cases. All peptides targeted at significantly (FDR=0.05) higher relative frequency in cases versus both adult and pediatric controls were derived from AAVs, except one Hepatitis B virus peptide. This peptide is a subsequence of the Hepatitis B virus Large Envelope Protein but shares two motifs with AAV peptides that were targeted at higher relative frequency in cases. The cytomegalovirus (CMV) and influenza peptides were not enriched in cases relative to the adult control group.

Many of the AAV peptides targeted in samples were from overlapping tiles from the same protein sequence or homologous regions of several *Parvoviridae* species (fig. S1 to 3). Targeting of overlapping peptides suggests that a linear epitope is located within the 28 amino acid stretch shared by the library peptides. Two distinct regions likely to contain epitopes were identified for the Rep68/78 protein (fig. S1 and 2), whereas a 224 amino acid stretch of 7 overlapping VirScan peptides that likely contains several linear epitopes was observed for the capsid VP1 protein (fig. S3)>. Targeting of homologous regions of other *Parvoviridae* species is likely evidence of a cross-reaction originating from AAV-2 immunity.

Among peptides targeted in all or most cases, these AAV peptides were remarkable in that they were targeted at very low frequency in all control groups (Fig. 2B). Most other peptides targeted at high frequency in cases contained previously identified “public epitopes,” or epitopes targeted frequently in seropositive individuals (12). These peptides were targeted in both the pediatric and adult control cohorts.

### Cases and controls cluster according to parvovirus reactivity.

**Fig. 3.**
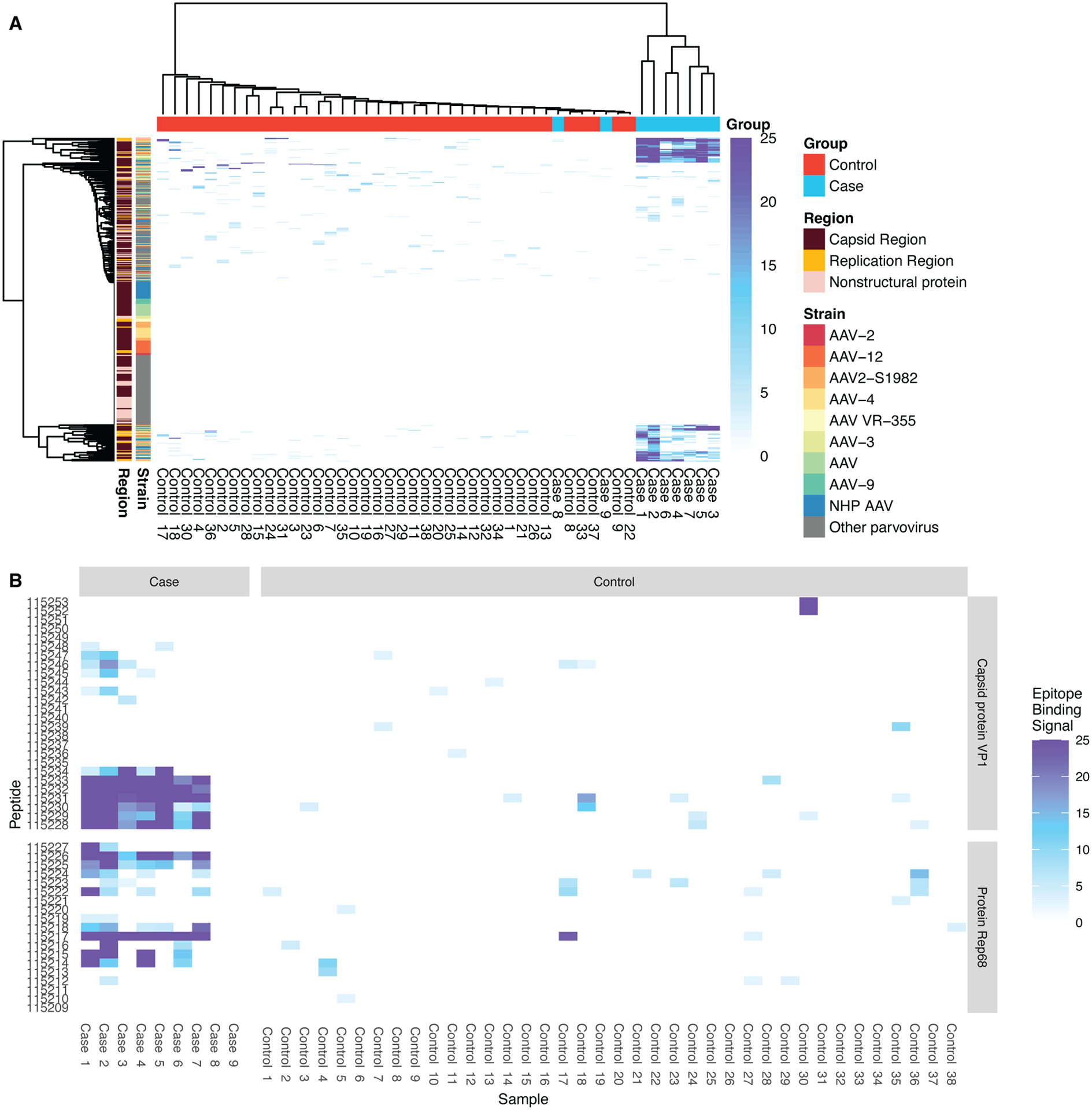
Antibody profiles of patients with AHUE clustered according to parvovirus reactivity and showed evidence of epitope spreading. (**A**) Shown is a heatmap of epitope binding signals for cases and pediatric controls for *Parvoviridae* peptides. Each row represents a VirScan library peptide and each column is a sample. Heatmap rows and columns are ordered according to complete agglomerative clustering with corresponding dendrograms shown. The strains and protein regions from which peptide sequences were derived is annotated along the y-axis and case-control status is annotated along the x-axis. (**B**) The heatmap shows the response to peptides derived from AAV-2 (isolate Srivastava/1982) protein sequences in cases and pediatric controls.

Complete hierarchical agglomerative clustering according to *Parvoviridae* reactivity clearly distinguishes the seven cases with any apparent AAV immunity from pediatric controls (Fig. 3A). Cases did not cluster together well when clustered according to *Adenoviridae* or *Herpesviridae* reactivity instead (fig. S4). Although adeno-associated dependoparvovirus reactivity was detected in pediatric controls, clustering by *Parvoviridae* reactivity appeared to be driven by a highly conserved and strong response to a set of specific AAV peptides from the capsid and replication region in cases with AAV immunity.

### Evidence of AAV-2 Epitope Spreading can be found in cases of AHUE.

All positive cases targeted several peptides derived from both the AAV-2 VP1 and AAV-2 Rep68 proteins. The average range of a linear epitope footprint is 4 to 22 amino acids (13–15). Thus, a single targeted 56-mer VirScan peptide could contain multiple distinct epitopes. When two adjacent peptides are recognized, a minimum of one epitope must exist. If only one epitope exists, it would be located in the overlapping 28-mer shared between the two peptides. If two recognized peptides are separated by a non-scoring peptide, antibodies in the sample bound a minimum of two epitopes. For example, for patient 1, there were a minimum of 7 distinct epitopes in VP1 and 5 in Rep68, demonstrating epitope spreading consistent with a robust antibody response (Fig. 3B).

AAV-2 positive cases targeted a mean of 40.4 ± 11.3 Adeno-associated dependoparvovirus A (AAV-A) VirScan peptides per child compared to means of 1.8 ± 0.8 and 1.0 ± 0.2 peptides for pediatric and adult controls, respectively. Homologous regions of other adeno-associated dependoparvoviruses were also targeted at high frequency in cases and at very low frequency or not at all in controls (figs. S1, S2).

### Strong AAV Antibody Responses were observed in serum from AHUE cases.

VirScan Epitope Binding Signal (EBS) correlates with antibody titer and can be used as a quantitative measure of strength of antibody response (16). To identify the strongest antibody responses in each sample, we rank-ordered VirScan peptides by VirScan EBS (from greatest to least) and examined the composition of the top 100 scoring peptides for each individual. Although AAV-A peptides only account for 0.15% of the VirScan library, they make up a mean of 6.4% ± 1.8% of the top 100 peptides rank-ordered by EBS in AHUE cases that target AAV-2 (N=7). In contrast, a single AAV-A peptide appears in the top 100 peptides rank-ordered by EBS for only one pediatric control (N=38).

### Immunity to AAV-2 Helper Viruses was enriched in cases.

We detected antibodies to HAdVs in all AAV-2 positive cases, consistent with previous clinical testing (6). We also detect immunity to at least two human herpesviruses (HHVs) in each of these cases (fig. S5A). Some HHVs were observed with greater frequency in cases than pediatric controls (fig. S5B).

## Discussion

In this study, we detected a conserved antibody response to specific regions of AAV-2 proteins in cases of AHUE that is not observed in pediatric or adult controls. Although associations between Adenovirus F viral detection and AHUE have been reported (6), we do not detect any differences in breadth of adenoviral antibody response or response to specific adenovirus peptides in cases versus controls. Because AAV-2 requires a helper virus to replicate and human adenoviruses can fulfill this role, it is possible that human adenovirus exposure is actually a lurking variable and associations between HAdVs and AHUE are spurious. This is supported by evidence that frequency of HAdV detection in the general AHUE population is only 45 to 52% (1, 3) and many of the cases negative for HAdVs tested positive for HHVs (17) which can also act as helper viruses for AAV-2 (18). Furthermore, during the course of the study, additional evidence of AAV-2 exposure in cases of AHUE was independently recorded in cohorts of AHUE patients in the United Kingdom(19, 20).

Notably, we have detected increased breadth of antibody response to AAVs in cases versus controls. Each case recognizes multiple regions within the AAV-2 capsid and replicase regions with evidence of epitope spreading. Moreover, these regions were conserved across AHUE cases but very rarely targeted in controls. This is not to say that AAV-2 is not targeted at all in the pediatric control group. Rather, the breadth of the AAV-A antibody response is far greater in AAV-2 positive cases than controls, with the seven AAV positive AHUE cases, each targeting 27 to 60 AAV-A peptides. No pediatric control targets more than 8 such peptides. About 15.4% of pediatric controls under 3 years old and 16.7% of those over three years old target at least 4 AAV-A peptides. These values are only slightly lower than previous AAV-2 seropositivity estimates of 21% in healthy children between 1 and 3 years old and 22% in children 3 to 18 years old (21). Part of this difference is due to the fact that VirScan slightly under detects prevalence for some viruses (e.g. measles) relative to enzyme-linked immunosorbent assays (ELISAs) in individuals with a past history of infection but readily detects recent infections (12, 16).

We also report a difference in magnitude of antibody responses to AAV-A in AAV positive AHUE cases relative to controls. Strong AAV-A responses account for a mean of 6.4 ± 1.8% of the top 100 peptides rank-ordered by VirScan EBS in AAV positive cases (N=7). Taken together, the strength and breadth of responses to AAV peptides in cases are consistent with peak antibody concentrations during infections and shortly after (22). This indicates recent infection with AAV-2.

We also detect immunity to HAdVs and HHVs, both of which can act as helper viruses to facilitate AAV-2 replication, in all AAV-2 positive cases. We detect immunity to at least two human herpesviruses in addition to adenovirus immunity in each of these cases. These findings are consistent with previously reported qPCR detection of human adenovirus and human herpesvirus DNA in samples from patients with AHUE (6). Presence of active infection with potential helper viruses and detection of AAV-2 immunity points to likely presence of replicating AAV-2 at the time of AHUE onset.

Our data reveals a strong correlation of AHUE with AAV-2 infections exhibiting a high titer and breadth of antibody responses indicative of a recent infection. These data implicate AAV-2 as a likely causative agent of the disease. If so, a central unanswered question is: Why is AAV-2, which infects many people, suddenly more pathogenic in cases of AHUE? Beyond the possibility that this variant is more pathogenic, one possible explanation is that coinfection with multiple helper viruses may act to increase the total number of cells infected with helper virus, and thus, the total number of cells in which AAV-2 replication is possible. This may contribute to increased viral titers, inflammation, and tissue damage leading to severe disease in a subset of AAV-2 infected children, a possibility that needs to be examined in additional cohorts. Consistent with this hypothesis, hepatotoxicity has been reported in high-dose AAV gene therapy trials, even leading to death (23). As a result, immunosuppressants are commonly co-administered with AAV-vectored gene therapy to prevent hepatotoxicity (24).

A speculative and complementary hypothesis that may play a role in emergence of AHUE concerns COVID-19 infection control measures and their potential to alter the timing of viral exposures. Years of masking prevents infection with many viruses at normal frequencies while immunity wanes for these viruses. Once masking was reduced, more viruses could have been in simultaneous circulation at higher frequencies in a more vulnerable population, possibly synchronizing infections. Thus, it is possible that as a result of these circumstances children may have been more likely to acquire multiple infections at once due to changes in masking policies and exposures than before the pandemic. This could increase the likelihood of concurrent infection with AAV-2 and one or more replicating helper viruses.

Seven patients in our cohort clearly had an immune response to AAV-2, but two did not. We do not believe that rules out AAV-2 as a causative agent of AHUE. Unexplained pediatric hepatitis is not a new phenomenon. Recent and historical cases of pediatric hepatitis with no identified cause are likely to stem from an array of biological mechanisms. It is possible that the two samples without AAV-2 have hepatitis driven by a different cause whereas the remaining seven may be part of a recent outbreak linked to AAV-2.

Our study has limitations. It is worth noting that due to sample availability, pediatric controls were not from the same area as cases and were not matched on demographic factors. Comparing cases to well-matched healthy controls from the same area would have been preferable and may have influenced results. Another limitation of this study is sample size. Determining whether the same patterns in AAV-2 antibody response are observed in independent AHUE cohorts would also be useful.

The distinct signatures of AAV-2 immunity detected in this cohort and the cases in Scotland are both elevated over infection frequency in controls. AAV-2 is therefore likely to be in some way responsible for AHUE. How this infection contributes to the development of AHUE mechanistically remains to be elucidated. Further research is needed to characterize the seroprevalence of AAV-2 antibodies in AHUE patients and identify mechanisms by which AAV-2 may be driving pediatric hepatitis onset.

## Methods

### Study Design

This is an observational case-control study utilizing previously collected serum samples from cases and several control groups as outlined below. The objective of this study was to identify whether there were differences in the antibody responses of cases of AHUE compared to controls with the goal of identifying a viral cause for AHUE. Sample size was determined by sample availability. Each serum sample was run on VirScan with two technical replicates to ensure that results were consistent between replicates. No samples were excluded from analysis. We observed the distributions of antiviral antibodies in cases and controls. Statistical analysis was used to evaluate differences between antibody responses of cases and controls overall and at the family, pathogen, and peptide levels.

### Sources of Serum

Secondary use of all human samples for the purposes of this work was exempted by the Brigham and Women’s Hospital Institutional Review Board (protocol number 2013P001337). Serum was obtained from nine patients under the age of 18 years admitted to Children’s of Alabama for severe acute hepatitis of unknown cause between October 1, 2021, and February 28, 2022. These samples were derived from patients previously characterized in a published case series (6). Samples from healthy children enrolled in the DIABIMMUNE study were used as pediatric controls. This cohort was described in previous studies (16, 25). The adult cohort was from a previous study designed to measure antibody responses in SARS-CoV-2 patients (26).

### VirScan

VirScan was performed using the VirScan 2.0 library (16) following a published protocol (27). In brief, VirScan is a Phage Immunoprecipitation Sequencing (PhIP-seq) technology. The library used in this study displays 115,753 peptides derived from published viral proteome sequences and known Immune Epitope Database (IEDB) epitopes on the surface of T7 bacteriophage. Diluted serum is incubated with the phage library and phage bound to antibodies are immunoprecipitated using Protein A/G coated magnetic beads. The phage insert DNA is then amplified and sequenced to determine which peptides were bound by antibodies in serum and to what degree. Epitope binding signals (EBS), a quantitative measure of antibody binding enrichment to each library peptide, and hits, a binary measure of whether a peptide was targeted or not were computed as previously described (16, 27). In brief, we consider a peptide recognized (“a hit”) if the EBS in both technical replicates is at least 3.5. EBS is presented as mean EBS across both technical replicates. EBS values below 0 have been artificially set to 0 for visualization.

### Data analysis

Overall antibody breadth was calculated as the total number of hits in a sample across the VirScan library. Hits are defined as peptides with epitope binding signals greater than 3.5 in both technical replicates. Pathogen-specific and family-wide breadth were calculated as the total number of hits across peptides derived from published protein sequences for a given pathogen species or family. Multiple sequence alignments were generated using Clustal Omega (28–30).

#### Statistical analysis

Figures were generated using R (version 4.1.2 with packages ggplot2 (31), ggpubr (32), ggmosaic (33), pheatmap (34), and ggplotify (35)), Adobe Illustrator, and UCSF ChimeraX (36). Statistical analysis was performed in R (37) (version 4.1.2). Welch’s T-test, Fisher’s Exact Test, and Benjamini-Hochberg adjusted p-values were computed using the t.test, fisher.test, and p.adjust functions respectively from the stats package (version 3.6.2). To test whether overall breadth of immune response was lower in cases versus controls, we used Welch’s one-sided t-test with an alpha of 0.05. To test whether immune breadth was higher in cases than controls within specific families and pathogens, Welch’s one-sided t-test was used with a Benjamini-Hochberg correction for multiple tests (FDR=0.05). In order to identify peptides targeted at significantly higher frequency in cases than controls, we used Fisher’s one-way Exact Test and adjusted for multiple tests with the Benjamini-Hochberg Procedure (FDR=0.05). Confidence intervals were constructed using 95% confidence intervals based on the t-distribution with the summarySE function in the Rmisc package (version 1.5.1).

## Supplementary Material

Supp. Text and Figures

Table S1

Table S2

Table S3

Table S4

Table S5

## Figures and Tables

**Table 1.** Demographic Characteristics of Cases and Controls.

	Cases (N=9)	Pediatric controls (N=38)	Adult controls (N=470)
**Age** – number (%)			
< 2 years	1 (11)	14 (37)	
2 to 5 years	5 (56)	24 (63)	
6 to 10 years	3 (33)		
11 to 17 years			
18 to 29 years			47 (10)
30 to 39 years			85 (18)
40 to 49 years			63 (13)
50 to 59 years			96 (20)
60 to 69 years			70 (15)
70 to 79 years			54 (11)
**> 80 years**			55 (12)
			
**Sex** – number (%)			
Male	2 (22)	20 (53)	228 (49)
Female	7 (78)	18 (47)	242 (51)
			
**Race/Ethnicity** – number (%)			
Non-Hispanic White	3 (33)	38 (100)	171 (36)
Hispanic White	6 (67)		82 (17)
Hispanic other			102 (22)
Non-Hispanic Black			39 (8)
Asian and Pacific islander			15 (3)
Non-Hispanic other			8 (2)
Hispanic Black			3 (1)
Unknown			50 (11)

## Data Availability

All data associated with this study are in the paper or supplementary materials. All reasonable requests for materials to the corresponding author will be fulfilled. The VirScan library is available from S.J.E. under a material transfer agreement with the Brigham and Women’s Hospital.
