# Supplementary material for: Signatures of AAV-2 immunity are enriched in children with severe acute hepatitis of unknown etiology
Source: Sci Transl Med. Author manuscript; Available in PMC 2023 Sep 14. (PMC10501808; doi:10.1126/scitranslmed.adh9917)
Supplement: Supp. Text and Figures [file NIHMS1929797-supplement-Supp__Text_and_Figures.docx]

**Supplemental Figures**

**Fig. S1. Structure and multiple sequence alignment of homologous amino acid sequences for adeno-associated virus (AAV-2) Rep epitope binding region 1.** The REP68_AAV2S epitope region 1 is highlighted in purple on the crystal structure (*38*) of the REP68 heptamer. Multiple sequence alignment is shown for the REP68_AAV2S epitope region and other VirScan peptides derived from homologous Rep proteins and enriched in cases versus controls. Each sequence is labeled with the UniProt Entry name, protein name, and organism from which the sequence was derived. REP68_AAV2S was used as the template sequence. Mismatches are indicated in red.

**Fig. S2. Structure and multiple sequence alignment of homologous amino acid sequences for AAV-2 Rep epitope binding region 2.** The REP68_AAV2S epitope region 2 is highlighted in purple on the crystal structure (*38*) of the REP68 heptamer. Multiple sequence alignment is shown for the REP68_AAV2S epitope region and other VirScan peptides derived from homologous Rep proteins and enriched in cases versus controls. Each sequence is labeled with the UniProt Entry name, protein name, and organism from which the sequence was derived. REP68_AAV2S was used as the template sequence. Mismatches are indicated in red.

**Fig. S3. Partial protein alignment of homologous *Parvoviridae* capsid sequences containing VirScan peptides that were enriched in cases versus controls.** Each sequence is labeled with the UniProt Entry name, protein name, and organism from which the sequence was derived. CAPSD_AAV2S was used as the template sequence. Mismatches are indicated in red. Bolded sequence regions indicate sequences represented in VirScan peptides that were observed at significantly (FDR=0.05) higher relative frequency in cases versus controls.

**Fig. S4. Heatmap of epitope binding signals for cases and pediatric controls for adenovirus (A) and herpesvirus (B) peptides.** Each row represents a VirScan library peptide and each column is a sample. Heatmaps rows and columns are ordered according to complete agglomerative clustering with corresponding dendrograms shown. The species from which the peptide sequence was derived is annotated along the y axis and the case-control status is annotated along the x-axis. EBS, Epitope Binding Signal.

**Fig. S5. Detection of helper virus in cases and pediatric controls.** (**A**) The heatmap shows predicted herpes virus seropositivity for cases and controls. Seropositivity was predicted using previously described virus score-based methodology (*16*). (**B**) The proportions of cases and controls that were seropositive for indicated herpes viruses are shown.

**Supplementary Data Files**

**Data File S1. Epitope binding signals for cases of acute hepatitis of unknown etiology (AHUE).** The ID number of the VirScan peptide and the pathogen species from which the peptide sequence was derived are given in the first two columns. The remaining columns contain the epitope binding signals for each case.

**Data File S2. Epitope binding signals for pediatric controls.** The ID number of the VirScan peptide and the pathogen species from which the peptide sequence was derived are given in the first two columns. The remaining columns contain the epitope binding signals for each control.

**Data File S3. Peptide hits for cases of AHUE.** The ID number of the VirScan peptide and the pathogen species from which the peptide sequence was derived are given in the first two columns. The remaining columns contain the hits for each case. Hits are denoted with “1.”

**Data File S4. Peptide hits for pediatric controls.** The ID number of the VirScan peptide and the pathogen species from which the peptide sequence was derived are given in the first two columns. The remaining columns contain the hits for each pediatric control. Hits are denoted with “1.”

**Data File S5. Peptide hits for adult controls.** The ID number of the VirScan peptide and the pathogen species from which the peptide sequence was derived are given in the first two columns. The remaining columns contain the hits for each adult control. Hits are denoted with “1.”
